# Small angle x-ray scattering with edge-illumination

**DOI:** 10.1038/srep30940

**Published:** 2016-08-05

**Authors:** Peter Modregger, Tiziana P. Cremona, Charaf Benarafa, Johannes C. Schittny, Alessandro Olivo, Marco Endrizzi

**Affiliations:** 1 Department of Medical Physics and Bioengineering, University College London, Gower Street, WC1E 6BT London, United Kingdom; 2 Institute of Anatomy, University of Berne, Baltzerstrasse 2, 3012 Bern, Switzerland; 3 Theodor Kocher Institute, University of Berne, Freiestrasse 1, 3012 Bern, Switzerland

## Abstract

Sensitivity to sub-pixel sample features has been demonstrated as a valuable capability of phase contrast x-ray imaging. Here, we report on a method to obtain angular-resolved small angle x-ray scattering distributions with edge-illumination- based imaging utilizing incoherent illumination from an x-ray tube. Our approach provides both the three established image modalities (absorption, differential phase and scatter strength), plus a number of additional contrasts related to unresolved sample features. The complementarity of these contrasts is experimentally validated by using different materials in powder form. As a significant application example we show that the extended complementary contrasts could allow the diagnosis of pulmonary emphysema in a murine model. In support of this, we demonstrate that the properties of the retrieved scattering distributions are consistent with the expectation of increased feature sizes related to pulmonary emphysema. Combined with the simplicity of implementation of edge-illumination, these findings suggest a high potential for exploiting extended sub-pixel contrasts in the diagnosis of lung diseases and beyond.

In small angle x-ray scattering sensitivity to sub-pixel sample features provides access to information on microscopic scales with macroscopic pixel sizes. In addition to complementary information about the sample this offers the opportunity for faster scans and/or dose reduction by exploiting larger pixel sizes. The ability to obtain contrasts related to sub-pixel sample structures was demonstrated with analyzer-based imaging^1^,^2^, grating interferometry (GI)^3^,^4^ and edge-illumination (EI)^5^,^6^. Currently, the potential of phase sensitive x-ray imaging for clinical application is investigated in the fields of mammography^7^,^8^,^9^, cartilage imaging^10^, bone structure determination^11^, imaging the airway surface liquid^12^ and the diagnosis of pulmonary emphysema^13^,^14^. Sub-pixel information is typically accessed through the broadening of the scattering distribution underlying the signal within a pixel, and is usually referred to as scatter strength. Scatter strength can be regarded as a measure for sample inhomogeneity within a detector pixel and its relation to sub-pixel sample morphology is the subject of ongoing research^15^,^16^,^17^,^18^,^19^.

In this study, we applied a deconvolution-based data analysis approach previously used in GI^20^,^21^ to EI, which increases the number of complementary contrasts related to sub-pixel sample features. EI is a non-interferometric and incoherent phase sensitive x-ray imaging technique^22^ utilizing a set of two apertured masks (Fig. 1). The pre-sample mask shapes the incident radiation into an array of well defined beamlets, which are distorted by the sample. These distortions are transformed into detectable intensity variations by the detector mask, which covers approximately half of each pixel. Since the optical elements are achromatic, EI is readily compatible with incoherent x-ray tubes, where the full spectrum of the source contributes to the signal at the detector^23^,^24^. The comparatively large structure sizes of the apertured masks (typically tens of microns) render EI easily scalable, robust against environmental vibrations and thermal stress. Established scan and data procedures simultaneously yield absorption contrast, differential phase contrast^25^,^26^ with nano-radiant sensitivity^27^ and scatter contrast^6^. Recently, EI was also successfully combined with tomography^28^,^29^.

## Extended Number of Contrasts with EI-SAXS

In the experiment, illumination curves (IC) were obtained by recording the detected intensity for each pixel while the sample mask was laterally scanned in *M* steps separated by a fraction of the mask pitch. This was performed with and without a sample present yielding the sample IC (*s*) and the flat IC (*f*), respectively. We express the ICs in terms of the scatter angle *α* (i.e., *s*(*α*) and *f*(*α*)), which is related to the lateral offset Δ*x* of the sample mask and the sample to detector distance *z* by *α* = Δ*x*/*z*. In analogy with the procedure described in^20^, we assumed that the distortion of the sample IC can be modelled by


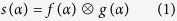


with the small-angle x-ray scattering (SAXS) distribution *g*(*α*) being determined by the sub-pixel structure of the sample. Therefore, *g*(*α*) can be retrieved by deconvolving the sample IC *s* with the flat IC *f*. In order to distinguish this analysis method from others we will refer to it as EI-SAXS.

For this proof of concept experiment, we used a dragon fly as a biological sample expected to provide a variety of differently shaped scattering distributions. During the experimental scan *M* = 10 images were acquired on equally spaced positions of the IC. Each image consisted of 5 frames with 5 s exposure time to ensure sufficient signal to noise ratio. In order to reduce possible aliasing effects due to partial illumination of the sample within one period of the sample mask, the sample was dithered 8 times (i.e., additional translations of the sample by a fraction of the sample mask pitch between the dithering steps were performed), and the corresponding images averaged prior to data analysis.

Data analysis was performed by deconvolving *s*(*α*) with *f*(*α*) using iterative Lucy-Richardson deconvolution^30^,^31^, where the *k*-th iteration is performed by computing


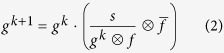


and 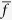 denotes *f* mirrored at the origin. In practice the occurring convolutions are computed utilizing fast Fourier transform. Lucy-Richardson deconvolution has an implicit positive constraint, converges to the maximum likelihood solution in the case of Poisson statistics, and is known to deliver stable results in the presence of photon shot noise. The starting value was chosen to be 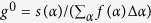, which provides the correct normalisation of the retrieved scattering distribution *g*. The number of iteration steps was 1000 in order to ensure convergence. The deconvolution provided the scattering distribution *g*(*α*) on the half-open interval 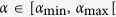. For the subsequent moment analysis (see below) it was convenient to symmetrize the scattering distribution (i.e., 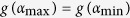), which is justified by the periodicity of the discrete Fourier transform employed by Lucy-Richardson deconvolution.

**Figure 2:**
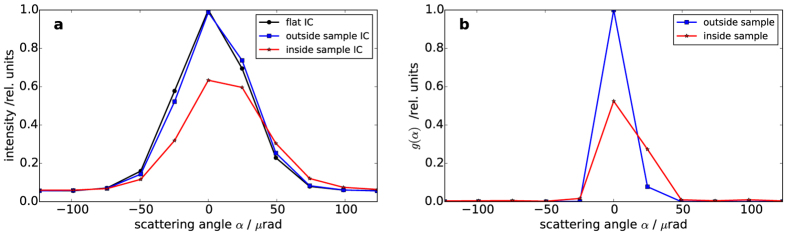
Recovery of scattering distributions. (**a**) Flat IC and sample ICs for a pixel outside and inside of the sample, respectively. (**b**) SAXS distribution as retrieved by deconvolution of the ICs shown in (**a**). As expected the retrieved distribution outside of the sample is approximately *δ*-shaped, while the distribution inside of the sample is shifted and broadened.

Figure 2 provides examples of the utilized ICs and of the retrieved scattering distributions inside and outside the sample. As expected, the scattering distribution outside the sample shows an approximate *δ*-function shape, where the finite width is due to finite sampling with respect to the scatter angle. The distribution inside of the sample is both shifted and broadened, which are both in accordance with expectations. Since the deconvolution procedure yields a scattering distribution *g*(*α*) for each pixel, scatter images (see Supplementary Figure 1) are available for each scattering angle *α*. This demonstrates an extension of the number of contrast from the previous 3 to 10 for EI. Ultimately, the number of contrast modalities is limited by the stability of the imaging system and by the resolution of the motor used for scanning the sample mask. Later in this study, we will utilize *M* = 32 sample points.

In GI, the retrieval of angular-resolved scattering distributions involves the deconvolution of noisy, periodic signals with suppressed even harmonics^20^, which is intrinsically a challenging task^32^. Moreover, owing to the coherence intrinsic to the contrast formation process in GI, parts of the broad spectrum provided by an x-ray tube contribute substantially less to the signal collected by a detector that integrates over photon energies^33^. In contrast, the ICs in EI are approximately Gaussian-shaped, and the achromaticity of the optical elements significantly improves the signal to noise ratio^24^. Thus, the deconvolution procedure is simpler for EI. However, due to the geometry of experimental set-ups angular sampling of the scattering distribution is about an order of magnitude larger for EI (Δ*α* = 25 *μ*rad here) than for GI (Δ*α* = 1.7 *μ*rad in^20^). Therefore, the two methods retrieve the scattering distribution on essentially complementary, and possibly expanding scales. For EI, finer angular sampling can be achieved by either increasing the distance between sample and detector or by increasing the number of acquired sample points *M* on the IC. The latter will be ultimately limited by the stability of the set-up and the resolution of utilized scanning motors.

### Complementarity of contrasts provided by EI-SAXS

Based on the assumption that the flat and the sample IC can be expressed as Gaussian functions, it was demonstrated that three contrast modalities (i.e., transmission *t*, refraction Δ*x*_*R*_ and scatter strength *σ*) can be retrieved from three experimental images^6^. In the following, we will use this established data analysis procedure for comparison. Please refer to the reference for more details.

The three contrast modalities can be regarded as parameters of the shape of the scattering distribution and can be retrieved from the deconvolved scattering distributions by calculating the moments *M*_*n*_ (with *n* ∈ N being the order of the moment) according to^34^


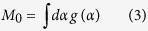



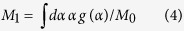



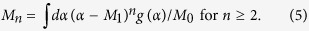


While *M*_0_, *M*_1_ and *M*_2_ correspond to absorption, differential phase and scatter strength contrasts, respectively, higher order moments (*n* > 2) constitute additional descriptive parameters for the scattering distribution and, thus, provide increased structural information about the sample on a sub-pixel scale.

**Figure 3:**
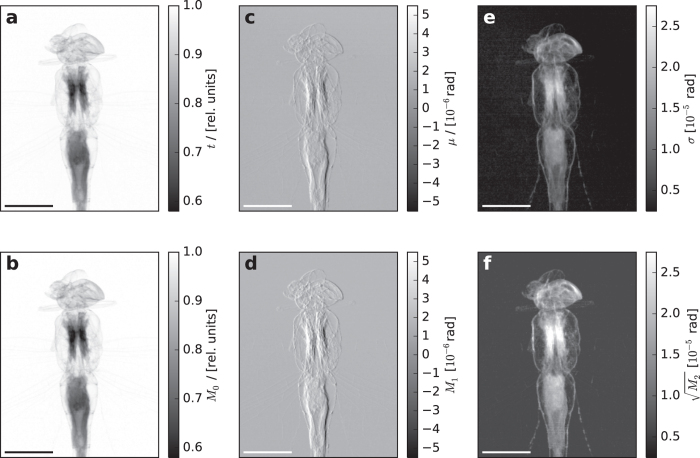
Comparison of an established data analysis procedure with the proposed deconvolution-based method for a dragon fly. The top row shows the transmission *t* (**a**), refraction *μ* (**c**) and scatter strength *σ* (**e**) retrieved according to^6^. The bottom row shows the first three moments of deconvolved scattering distribution: (**b**) *M*_0_ corresponds to transmission, (**d**) *M*_1_ to refraction and (**f**) *M*_2_ to scatter strength, respectively. Scale bars are 1 cm. An excellent agreement between *M*_0_ and *M*_1_ and their corresponding contrasts retrieved from the established data analysis was found. 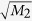 and *σ* show the same sample morphology while slightly differing in the magnitude of retrieved values (factor of ≈1.5). The deviation is attributed to a violation of the strict assumptions of the previously established data analysis.

Figure 3 shows a comparison of different contrast modalities as retrieved by the established data analysis to the corresponding moments of deconvolved scattering distributions at the example of a dragon fly. We found an excellent agreement for *M*_0_ and the transmission *t* (correlation coefficient: 0.999) as well as for *M*_1_ and the refraction contrast Δ*x*_R_ (correlation coefficient: 0.954). *M*_2_ and the scatter strength *σ* show the same sample morphology (correlation coefficient: 0.926), while they differ only in the magnitude of the retrieved scatter strength. This deviation can attributed to the fact that the shape of the sample and flat IC differ from a Gaussian, which leads the established data analysis to wrongly estimate the scatter strength.

This establishes EI-SAXS as a viable alternative to the data analysis methods previously used in imaging with edge-illumination. It should be noted that, while previously reported data methods made assumptions about the underlying scattering distribution (e.g., negligible scattering in^25^,^26^), the proposed deconvolution method does not assume a specific shape of the involved IC and, thus, produces reliable results for arbitrarily shaped scatter distributions.

**Figure 4:**
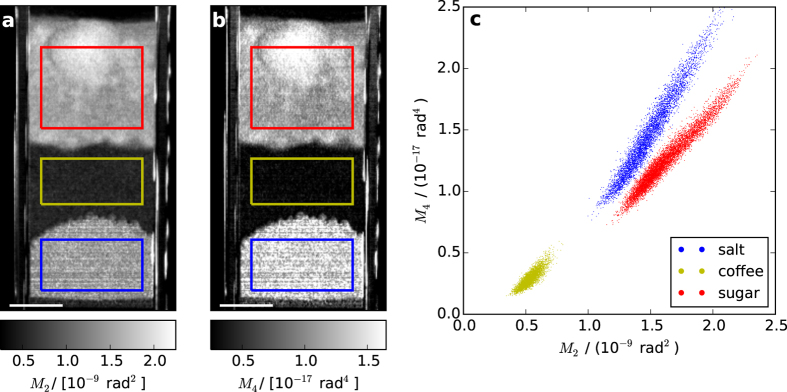
Complementary of moments extracted from different powders with EI-SAXS. *M*_2_ (**a**) and *M*_4_ (**b**) for salt (blue rectangle), coffee (yellow rectangle) and sugar (red rectangle). Scale bars are 5 mm. The pixel-wise scatter plot of *M*_2_ and *M*_4_ (**c**) demonstrates the complementarity of those moments.

In order to investigate the information content provided by higher order moments we investigated the scattering properties of three household powders: salt, coffee and sugar. The same parameters for the scan and analysis procedures as above were used, except for the lack of dithering (i.e, the sample was not moved). Figure 4(a,b) show the 2^nd^ and 4^th^ moment of the scattering distributions, respectively retrieved by deconvolution. The complementarity of *M*_2_ and *M*_4_ is clearly demonstrated by the corresponding pixel-wise scatter plot, shown in Fig. 4(c). Therefore, EI-SAXS provides the opportunity to exploit extended sub-pixel contrasts, which could be useful in areas as diverse as biomedical research, materials science and security screening. As an example of a significant application, we will take a first step into exploring the potential of EI-SAXS for the diagnosis of lung diseases.

### EI-SAXS for the diagnosis of pulmonary emphysema

Chronic obstructive pulmonary disease (COPD) is characterized by severe changes in lung morphology and is a major and increasing cause of death associated with cigarette smoking^35^. The micro-structure of lungs is defined by alveolar walls and distal airspaces, which have typical sizes on the micrometer scale^36^. Pulmonary emphysema is a frequent constituent of COPD and a condition of the lung “characterized by abnormal, permanent enlargement of air spaces distal to the terminal bronchiole, accompanied by destruction of their walls, and without obvious fibrosis”^37^.

In the following pilot experiment with a mouse model of cigarette smoke exposure-induced emphysema, we demonstrate that EI-SAXS can detect this increase in feature sizes by exploiting the complementarity of the second and fourth moment of deconvolved scattering distributions. Two murine lungs, one control (N = 1) and one emphysematous (N = 1) were imaged. The same scan parameters and data analysis procedures as described above were used except for the number of sample points on the IC (*M* = 32) and the fact that dithering was not performed. Images for the *M*_0_, *M*_2_ and *M*_4_ can be found in the Supplementary material (supplementary Figure 2).

**Figure 5:**
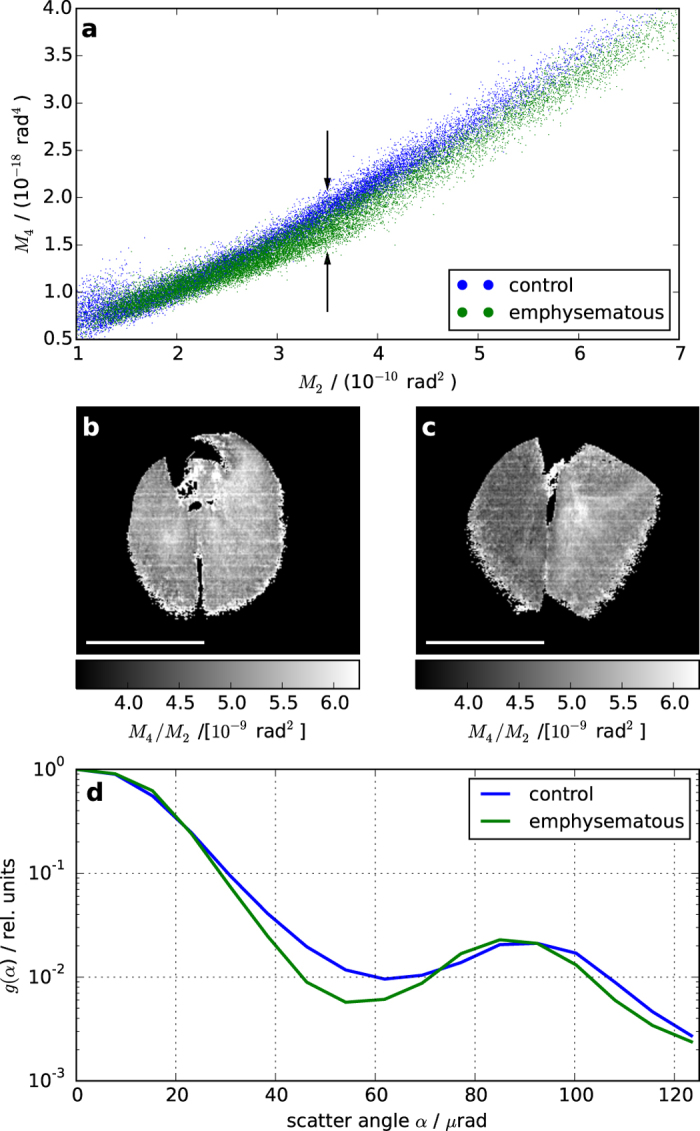
Diagnosis of lung emphysema with EI-SAXS. A clear separation of the control and the emphysematous lung in the *M*_2_–*M*_4_ scatter plot (**a**) is indicated by arrows. The ratio *M*_4_/*M*_2_ of the control (**b**) is larger than that of the emphysematous lung (**c**), which is most noticeable in the emphysematous left lung. Scale bars are 1 cm and the background was masked by thresholding. Comparison between scattering distributions (**d**) confirm that the emphysematous lung provides features smaller tails than that of the control one, which is consistent with increased average feature sizes in the former.

The scatter plot of *M*_2_ and *M*_4_ (Fig. 5a) reveals a clear distinction between control and emphysematous lung. Due to the small number of samples, we can only consider this as preliminary evidence for the utility of higher order moments provided by EI-SAXS in the context of pulmonary emphysema diagnosis. Further, for each lung sample an approximate linear relationship between *M*_2_ and *M*_4_ was found (also visible for the powders in Fig. 4c). Based upon this observation, the ratio of the 4^th^ and the 2^nd^ moment (i.e., *M*_4_/*M*_2_), which appears as the slope of lines connecting the plotted points to the origin, was used as an illustration model for the potential diagnostic power of EI-SAXS. The physical interpretation of this ratio is as follows. For a constant *M*_2_, larger values of *M*_4_ indicate stronger tails of the scattering distribution (see equation 5). In turn, stronger tails indicate a larger contribution of large scattering angles to the scattering distribution compared to small scattering angles. Since large scattering angles are associated with smaller sample features, high values of the *M*_4_/*M*_2_-ratio are related to larger relative contributions of smaller sample features. Potentially, the other moments (i.e., *M*_3_,*M*_5,…_) could carry additional information about the sub-pixel sample morphology. However a detailed analysis of their possible contributions lies outside the scope of this preliminary study and will be the subject of future investigations.

The images of *M*_4_/*M*_2_-ratios for the control and the emphysematous lung (Fig. 5b,c) appear generally flat and the emphysematous lung has noticeably darker appearance–especially in the left lung. Faint horizontal stripes are residuals from bridges in the mask design that stabilize the structure^38^. These minor artefacts can be easily removed by e.g. improved mask design or data analysis. The flatness of the *M*_4_/*M*_2_-contrasts is remarkable for two reasons. First, due to the opening in the sample mask only 10 *μ*m/79 *μ*m ≈ 12.7% of the sample was illuminated within a pixel (no dithering was used in this case) indicating the robustness of *M*_4_/*M*_2_-ratio. Second, sample movement does affect but not destroy a quasi-homogeneous contrast modality as it was shown with breathing during dark-field imaging of mouse lungs^13^. Since *M*_4_/*M*_2_ is homogeneous we speculate that breathing during exposure should only have a minor impact on EI-SAXS, as has been observed in the case of GI.

The mean *M*_4_/*M*_2_-ratios for the two lungs, determined over the entire sample, were 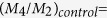 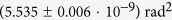 and 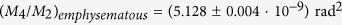, where the uncertainties are given at 0.68 confidence interval (CI). Thus, the two ratios were separated by more than 40 CIs. While the specific values of *M*_4_/*M*_2_ could potentially be affected by variations in experimental parameters, this very significant separation suggests a high potential of EI-SAXS for reliable diagnosis of emphysema at different stages of development.

The scattering distributions in Fig. 5(d) confirmed that the tails of the control were stronger than the tails of the emphysematous lung. Since smaller scattering angles are associated with larger sample features, the smaller tails of the diseased lung are consistent with increased average feature sizes due to emphysema. Since the higher order moments correspond to scattering of sub-pixel sample features, the pixel size could be increased without losing access to this information.

**Figure 6:**
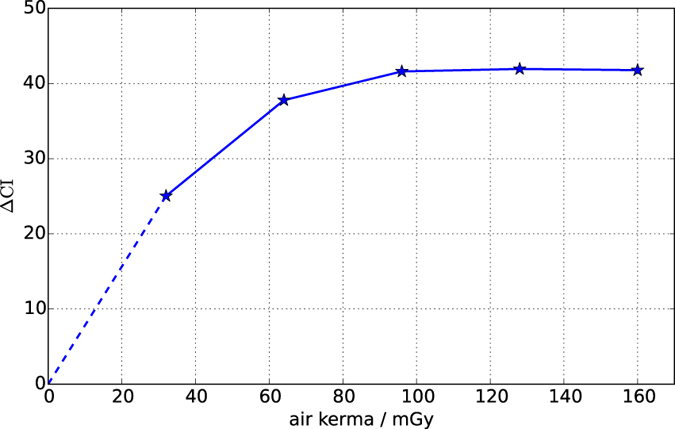
CI separation (Δ*CI*) of *M*_4_/*M*_2_ values for the control and the emphysematous lung as a function of delivered dose. The dashed line represents a linear extrapolation.

In this proof of concept study no special attention was paid to dose optimization. Using an ion chamber the dose rate was estimated as 0.2 mGy/s entrance air kerma. This implies a delivered dose of 400 mGy for the dragon fly (Fig. 3), 50 mGy for the powders (Fig. 4) and 160 mGy for the lungs (Fig. 5). However, the dose can be significantly reduced by optimizing aperture sizes^8^, adequate filtering of low energy photons^29^ and increasing pixel sizes. In order to demonstrate this potential, we simulated experiments with decreased dose by using 1, 2, 3, 4 or 5 out of the 5 accumulations that were acquired for the murine lungs as the input for data analysis. Figure 6 shows the resulting separation of the *M*_4_/*M*_2_-ratio for the control and the emphysematous lung in terms of CIs as a function of simulated dose. It is obvious that the separability of healthy and emphysematous lungs is preserved if the dose is reduced significantly. If a separation of 5 CIs is taken as sufficient, then a rough linear extrapolation (dashed line in Fig. 6) implies a dose of only 6.3 mGy with this un-optimized set-up. Thus, EI-SAXS offers the potential to diagnose pulmonary emphysema at low dose.

## Discussion

We presented a deconvolution based method for x-ray imaging based on the edge-illumination principle that provides the angular resolved small angle x-ray scattering distribution with a laboratory-based set-up (EI-SAXS). EI-SAXS makes no prior assumptions about the shape of the scattering distributions, and it was shown to extend the number of contrast modalities from previously three to ten and more. Using different household powders we demonstrated the complementarity of the 2^nd^ and 4^th^ moment of retrieved scattering distributions, which indicates access to additional information about sub-pixel sample features. We provided preliminary evidence for the possibility to exploit this complementarity in order to differentiate between healthy and emphysematous mouse lungs. We also demonstrated that the properties of experimental scattering distributions were consistent with larger average feature sizes in the emphysematous lung. Based on these observations, we introduced the ratio of the 4^th^ and 2^nd^ moment as an illustration model for emphysema diagnosis. Although the number of samples was small, the observed effect was very large, with the *M*_4_/*M*_2_-ratios separated by more than 40 CIs. In combination with the simple translatability of edge-illumination to a commercial system, the achieved results indicate EI-SAXS’s high potential for the exploitation of additional sub-pixel contrasts in biomedical research, materials science and medical diagnostics - especially for, but not limited to, improving the detection and characterisation of pulmonary emphysema.

## Methods

### Sample preparation

The dragon fly was air-dried over several days prior to scanning in order to ensure stable sample features and fixed at its tail for imaging. The three household powders were put on top of each other in a rectangular plastic holder to ensure consistent sample thicknesses. Wild-type 129S6/SvEv/Tac mice were exposed to air or to cigarette smoke 5 hours/day, 5 days/week for 4 months. Smoke of 3R4F research cigarettes (University of Kentucky, Lexington, KY) was generated by a TE10z smoking machine (Teague Enterprises, Woodland, CA) connected to whole-body exposure chambers as described previously^39^. All animal studies were approved by the Cantonal Veterinary Office of Bern, Switzerland and carried out in accordance with relevant guidelines. Lungs were instilled with 1.5% paraformaldehyde-1.5% glutaraldehyde in 0.15 M HEPES pH 7.35 through a tracheal cannula at a constant pressure of 20 cm H_2_O. The trachea was ligated and lungs were placed in fresh fixative for at least 24 hours to complete fixation. Following super-critical drying, samples were placed in the x-ray system using sample holder with membranes for imaging.

## Instrumentation

The experiment was carried out with a laboratory-based set-up at University College London (London, UK). The source was a Rigaku MM007 microfocus rotating anode with a Mo target operated at 25 mA current and 40 kVp voltage. A Hamamatsu C9732DK flat panel sensor was used as a detector featuring an isotropic pixel size of 50 *μ*m. Both the sample and detector mask were manufactured by Creatv Microtech (Potomac, MD) and consisted of a series of Au lines on a graphite substrate. The sample mask featured a pitch of 79 *μ*m and an opening of 10 *μ*m, while the detector mask had a pitch of 98 *μ*m and an opening of 17 *μ*m to account for beam divergence. In order to compensate for the comparatively large cross-pixel talk of the x-ray camera (≈40% for nearest neighbours), both masks had a column-skipped design^38^ and the raw images were binned 2 times for subsequent data analysis (effective pixel size 100 *μ*m). The sample to detector distance was *z* = 0.32 m and the total set-up length amounted to 2 m.

## Additional Information

**How to cite this article**: Modregger, P. *et al.* Small angle x-ray scattering with edge-illumination. *Sci. Rep.* **6**, 30940; doi: 10.1038/srep30940 (2016).

## Supplementary Material

Supplementary Information

## Figures and Tables

**Figure 1:**
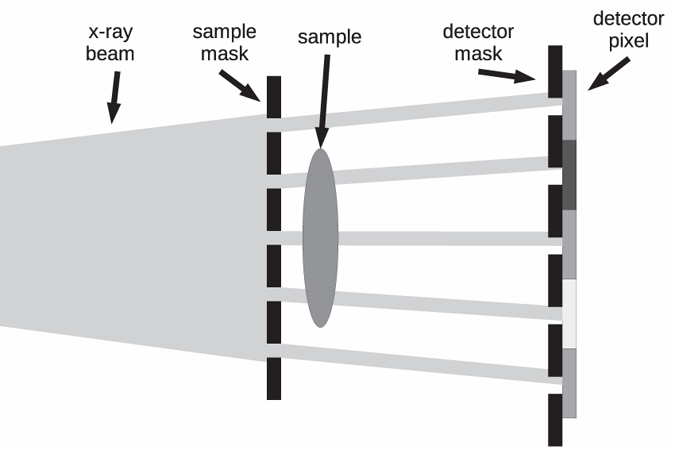
Sketch of the experimental set-up for EI.
