# Supplementary material for: Small angle x-ray scattering with edge-illumination
Source: Sci Rep. 2016 Aug 5;6:30940. doi: 10.1038/srep30940 (PMC4974648; doi:10.1038/srep30940)
Supplement: Supplementary Information [file srep30940-s1.pdf]

Small angle x-ray scattering with edge-illumination

Peter Modregger,[1] Tiziana P. Cremona,[2] Charaf Benarafa,[3]
Johannes Schittny,[2] Alessandro Olivo,[1] and Marco Endrizzi[1]

[1] *Department of Medical Physics and Bioengineering, University College London, Gower Street, WC1E 6BT London, United Kingdom*
[2] *Institute of Anatomy, University of Berne, Baltzerstrasse 2, 3012 Bern, Switzerland*
[3] *Theodor Kocher Institute, University of Berne, Freiestrasse 1, 3012 Bern, Switzerland*

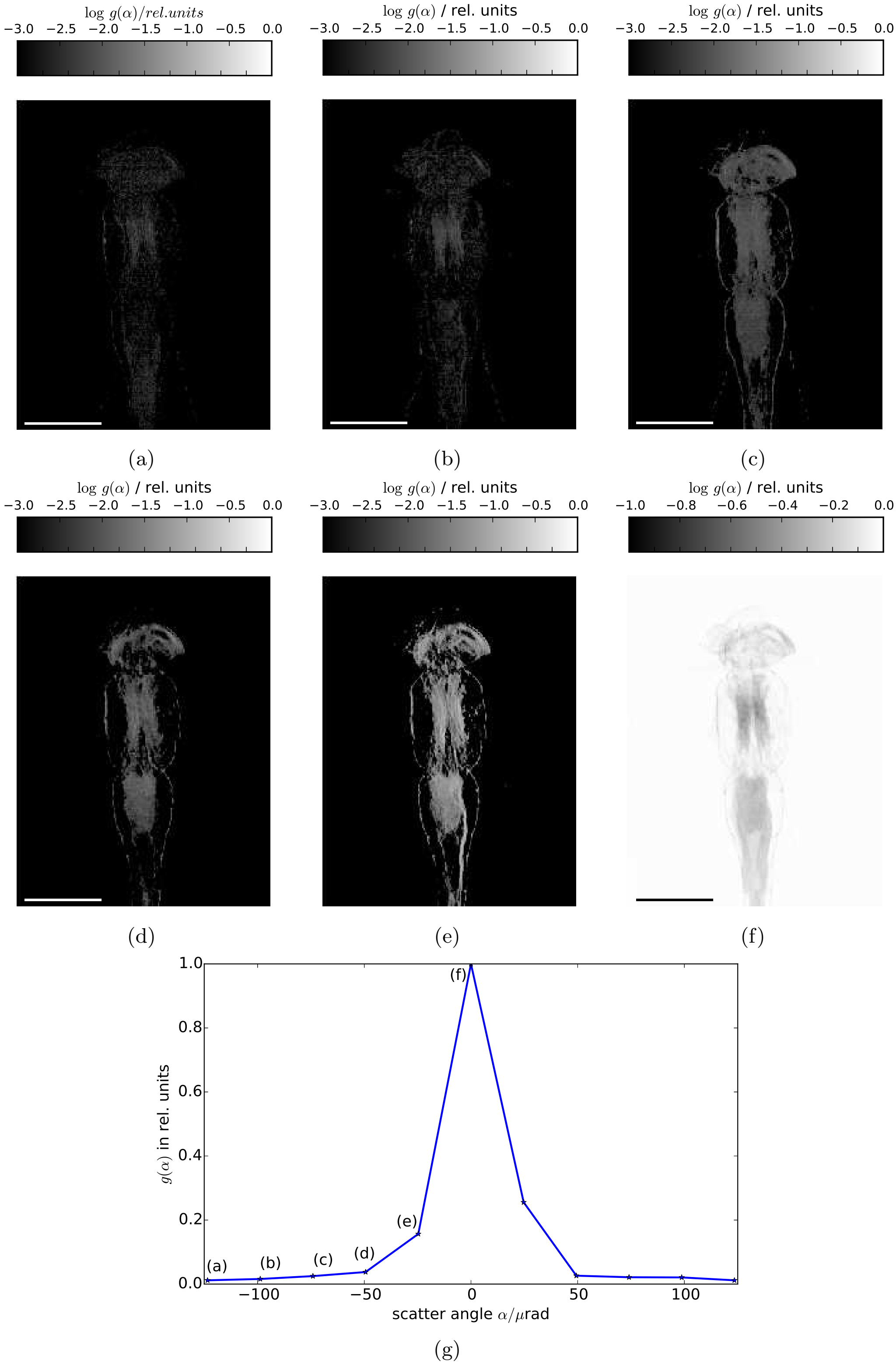

Supplementary Fig. 1: Scatter images of a dragon fly. (g) indicates the position of the individual scatter images on the global scattering curve. Scale bars are 1 cm.

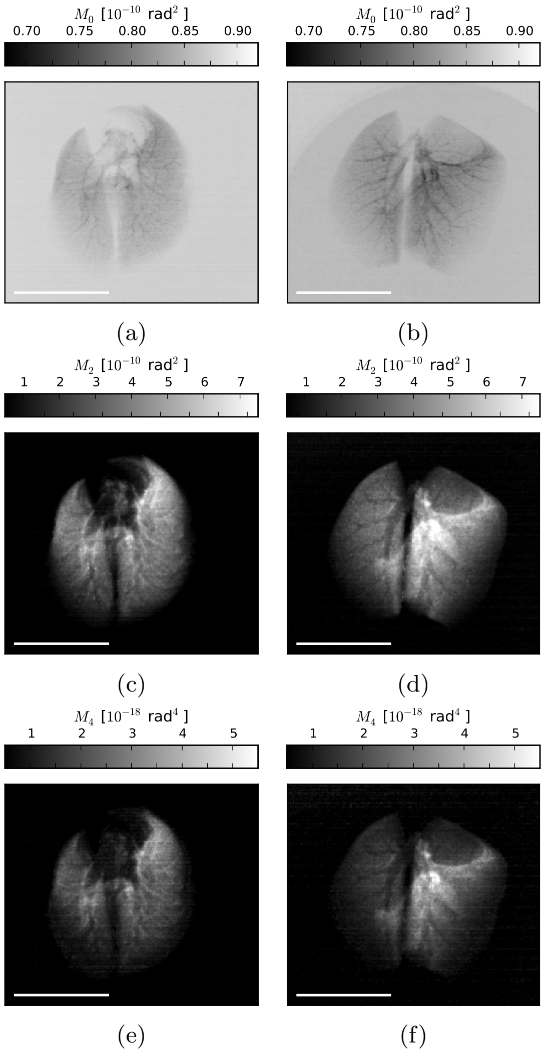

Supplementary Fig. 2: Different moments provided by EI-SAXS for mouse lungs. M_0 (a-b), M_2 (c-d) and M_4 (e-f) for the control sample (a,c,e) and the emphysematous sample (b,d,f). Scale bars are 1 cm.
